# Quantitative assessment of myocardial motion from displacement measurements derived from velocity encoded MRI

**DOI:** 10.1186/1532-429X-14-S1-W20

**Published:** 2012-02-01

**Authors:** Anja Lutz, Jan Paul, Patrick Etyngier, Axel Bornstedt, Gerd Ulrich Nienhaus, Peter Bernhardt, Wolfgang Rottbauer, Volker Rasche

**Affiliations:** 1 University Hospital of Ulm, Ulm, Germany; 2 Institute of Applied Physics and Center for Functional Nanonstructures, Karlsruhe Institute of Technology, Karlsruhe, Germany; 3 Medisys Research Lab, Philips Healthcare, Suresnes, France

## Summary

It is objective of this study to investigate the potential of velocity encoded MRI (TPM) to derive quantitative parameters based on local displacement information for the automatic assessment of mechanical asynchrony in CRT patients.

## Background

About 30% of patients treated with cardiac resynchronization therapy (CRT) do not benefit. Several MRI derived parameters have been published for quantification of mechanical asynchrony. Most parameters are based on tagging techniques. Evaluation of the tagging information is often cumbersome due to fading of the tag pattern (SPAMM) or low SNR (CSPAMM) especially during diastole.

## Methods

12 volunteers (30±8 years) and 3 patients (41±11 years) were investigated at a 3T whole body MR scanner (Achieva, Philips) with a 32 channel coil. The patients suffer from DCM, asynchrony and/or LBBB. A velocity encoded navigated segmented gradient echo sequence was applied in the apical, equatorial and basal slice. Acquisition parameters: FOV=340^2^mm^2^, in-plane res. =2.5^2^mm^2^, slice thickness =8mm, TR/TE=6.3ms/4.6ms, α=15°, 3 k-lines per segment, VENC=30cm/s,scan duration =5.51 min., black blood imaging with alternating presaturation pulses[1], SENSE=2 and 32 cardiac phases for 60 bpm.

Displacement and strain were calculated from the TPM data. Quantitative parameters derived included: circumferential and radial temporal uniformity of strain (TUS)[2-5], regional variance of strain (RVS) and regional variance of principle strain(RVVPS)[2], standard deviation of onset and peak time (SD(T_onset_),SD(T_peak_))[6], coefficient of variance (CV) and difference between septal and lateral peak circumferential strain (DiffSLpeakCS)[6,7], the absolute values of the OS and PS delay vector between septal-lateral (SL), inferior-anterior (IA) and apical-basal (AB) wall[8,9] and the base apex rotation coefficient (BARC)[10].

## Results

**Figure 1:**
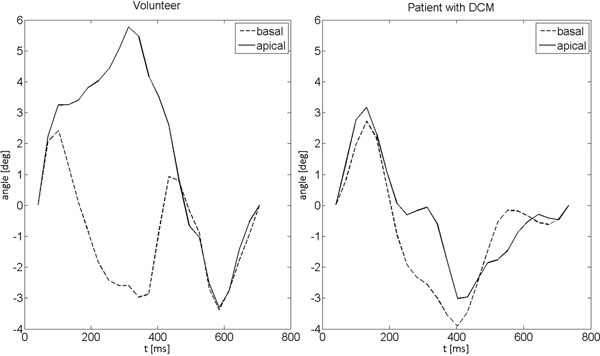
Circumferential displacement in the apical and basal slice show twistin in healthy volunteers, whereas this twistin motion is reduced in the patient with DCM.

Table 1 summarizes the quantified parameters for the volunteer and patient group. TUS_c_, SD(Tonset), the coefficient of variation, all OS delay values and the SL and IA PS delay values and BARC are not within the standard deviation of the respected volunteer data. Figure 1 shows the displacement of apical and basal slices exemplarily for one volunteer and one patient with proven asynchrony, with a clear loss of twisting motion.

**Table 1:** Displacement and strain derived motion quantification parameters calculated from tissue phase mapped MRI data are displayed as mean ± standard deviation in volunteers and for all three investigated patients.

parameter	volunteers		patients	
	mean	σ	mean	σ

TUS_c_	0.89	0.03	0.74	0.03
TUS_r_	0.86	0.04	0.76	0.07
RVS_max_ [%^2^]	62.22	30.68	114.49	57.90
RVVPS_max_ [%]	88.45	28.33	145.34	57.29
SD(T_onset_)[ms]	15.18	4.28	34.42	14.12
SD(T_peak_)[ms]	39.53	8.52	60.32	21.34
CV [%]	27.89	10.77	47.15	21.13
DiffSLpeakCS [%]	0.14	2.76	1.80	0.97
OS delay SL [ms]	0.77	1.88	52.11	1.88
OS delay IA [ms]	2.10	6.04	19.86	6.04
OS delay AB [ms]	3.63	12.58	41.63	12.58
PS delay SL [ms]	28.53	21.06	195.77	21.06
PS delay IA [ms]	9.68	9.46	158.01	9.46
PS delay AB [ms]	58.30	34.30	86.32	34.30
BARC	-0.02	0.32	0.66	0.16

## Conclusions

All parameters for asynchrony quantification based on tagging published earlier can also be derived from TPM data and similar differences between volunteers and patients as known from tagging are observed. Since TPM data provide higher spatial resolution and the evaluation appears less error prone than the tagging analysis, TPM may become a helpful adjunct for automatic assessment of mechanical asynchrony.

## Funding

AL and VR have a research agreement with Philips Medical, PE is employed by Philips Healthcare.
